# Supplementary material for: The Association between ADHD and Environmental Chemicals—A Scoping Review
Source: Int J Environ Res Public Health. 2022 Mar 1;19(5):2849. doi: 10.3390/ijerph19052849 (PMC8910189; doi:10.3390/ijerph19052849)
Supplement: Supplementary file 1 [file ijerph-19-02849-s001.zip › ijerph-1576914-supplementary.pdf]

Moore S et al. The Association between ADHD and Environmental Chemicals - A Scoping Review.

Supplementary material

Supplementary tables of individual papers included in each systematic review discussed in current paper. Studies that have been included in multiple reviews on the same substance have been underlined.

Table S1. Individual papers included in each systematic review providing evidence of moderate-high association between ADHD and each substance.

**Substance**	**Lead (Pb)**		**Phthalates**		**Bisphenol A (BPA)**	
**Systematic review**	Donzelli et al., 2019 (n=17)	Nilsen & Tulve, 2020 (n=13)	Praveena et al., 2020 (n=16)	Nilsen & Tulve, 2020 (n=2)	Nilsen & Tulve, 2020 (n=3)	Rochester et al., 2018 (n=3)
**Individual papers included in each systematic review**<br><br>(first author, year)	Huang 2016<br>Zhang 2015<br>Joo 2017<br>Park 2016<br>Ji 2018<br>Choi 2016<br>Neugebauer 2014<br>Forns 2014<br>Sioen 2013<br>Yang 2018<br>Lee 2018<br>Yu 2016a<br>Yu 2016b<br>Chan 2015<br>Hong 2015<br>Kim 2013<br>Dikme 2013	Huang 2016<br>Zhang 2015<br>Joo 2017<br>Park 2016<br>Kim 2010<br>Liu 2014<br>Nicolescu 2010<br>Nigg 2010<br>Roy 2009<br>Wang 2008<br>Yousef 2011<br>Boucher 2012<br>Ha 2009	*Cross-sectional*<br>Arbuckle 2016a<br>Won 2016<br>Park 2015<br>Chopra 2014<br>Kim 2009<br><br>*Cohort*<br>England-Mason 2020<br>Ku 2020<br>Balalian 2019<br>Minatoya 2018<br>Messerlian 2017<br>Verstraete 2016<br>Lien 2015<br>Kobrosly 2014<br>Engel 2010<br><br>*Case-control*<br>Engel 2018<br>Hu 2017	Hu 2017<br>Won 2016	Harley 2013<br>Li 2018<br>Tewar 2016	Harley 2013<br>Casas 2015<br>Braun 2011

Table S2. Individual papers included in each systematic review providing evidence of limited association between ADHD and each substance.

**Substance**	**Polycyclic aromatic hydrocarbons (PAHs)**		**Flame retardants**		**Mercury (Hg)**	
**Systematic review**	Rezaei Kalantary et al., 2020 (n=6)	Aghaei et al., 2019 (n=7)	Lam et al., 2017 (n=9)	Doherty et al., 2019 (n=2)	Yoshimasu et al., 2014 (n=2)	Nilsen & Tulve, 2020 (n=3)
**Individual papers included in each systematic review**<br><br>(first author, year)	Abid 2014<br>Mortamais 2017<br>Perera 2018<br>Perera 2014<br>Perera 2012<br>Perera 2011	Abid 2014<br>Mortamais 2017<br>Perera 2018<br>Perera 2014<br>Perera 2012<br>Perera 2011<br>Margolis 2016	Adgent 2014<br>Chen 2014<br>Cowell 2015<br>Eskenazi 2013<br>Gascon 2011<br>Gump 2014<br>Hoffman 2012<br>Roze 2009<br>Sagiv 2015	Castorina 2017<br>Doherty 2019	*Environmental Hg*<br>Sagiv 2012<br>Boucher 2012<br><br>(Environmental methyl mercury with relevant exposure periods)	Ha 2009<br>Yousef 2011<br>Boucher 2012

**Substance**	**Pesticides**			
**Systematic review**	Nilsen & Tulve, 2020 (n=6)	Roberts et al., 2019 (n=12)	Tessari et al., 2020	
**Individual papers included in each systematic review**<br><br>(first author, year)	*Organic contaminants (OC)*<br>Bouchard 2010a<br>Quiros-Alcala 2014<br>Wagner-Schuman 2015<br>Xu 2011<br>Yu 2016a<br>Newman 2014	*ADHD + pesticides*<br>Bouchard 2010<br>Quiros-Alcala 2014<br>Wagner-Schuman 2015<br>Yu 2016<br>Van Wendel de Joode 2016<br>Marks 2010<br>Sagiv 2010<br>Sioen 2013<br>Saez 2018<br>Rauh 2015<br>Ribas-Fito 2007<br>Richardson 2015	*ADHD + OPs (n=8)*<br>Bouchard 2010<br>Yu 2016<br>Van Wendel de Joode 2016<br>Marks 2010<br>Eskenazi 2007<br>Fortenberry 2014<br>Oulhote 2013<br>Rauh 2006	*ADHD + OC/PCB (n=5)*<br>Sagiv 2010<br>Sioen 2013<br>Xu 2011<br>Forns 2018<br>Lenters 2019<br><br>*ADHD + pyrethroid (n=3)*<br>Quiros-Alcala 2014<br>Wagner-Schuman 2015<br>Oulhote 2013

Table S3. Individual papers included in each systematic review providing evidence of low association between ADHD and each substance.

Substance	Cadmium (Cd)	Per- and polyfluoroalkyl compounds (PFAS)	
**Systematic review**	Sanders et al., 2015 (n=5)	Lee YJ et al., 2018 (n=5)	Nilsen & Tulve, 2020 (n=2)
**Individual papers included in each systematic review**<br><br>(first author, year)	Forns 2014<br>Szkup-Jablonska 2012<br>Kim S 2013<br>Yousef 2011<br>Ciesielski 2013	Lien 2016<br>Stein & Savitz 2011<br>Hoyer 2015<br>Liew 2015<br>Ode 2014*<br><br>*Not included in table in Lee YJ, 2018, but included in text segment	*Perfluorinated compounds (PFC)*<br>Hoffman 2010<br>Stein 2014

Systematic reviews included in current paper

Aghaei M, Janjani H, Yousefian F, Jamal A, Yunesian M. Association between ambient gaseous and particulate air pollutants and attention deficit hyperactivity disorder (ADHD) in children; a systematic review. Environ Res. 2019 Jun;173:135-156. doi: 10.1016/j.envres.2019.03.030. Epub 2019 Mar 15. PMID: 30909100.

Doherty BT, Hammel SC, Daniels JL, Stapleton HM, Hoffman K. Organophosphate Esters: Are These Flame Retardants and Plasticizers Affecting Children's Health? Curr Environ Health Rep. 2019 Dec;6(4):201-213. doi: 10.1007/s40572-019-00258-0. PMID: 31755035; PMCID: PMC8631201.

Donzelli G, Carducci A, Llopis-Gonzalez A, Verani M, Llopis-Morales A, Cioni L, Morales-Suárez-Varela M. The Association between Lead and Attention-Deficit/Hyperactivity Disorder: A Systematic Review. Int J Environ Res Public Health. 2019 Jan 29;16(3):382. doi: 10.3390/ijerph16030382. PMID: 30700018; PMCID: PMC6388268.

Lam J, Lanphear BP, Bellinger D, Axelrad DA, McPartland J, Sutton P, Davidson L, Daniels N, Sen S, Woodruff TJ. Developmental PBDE Exposure and IQ/ADHD in Childhood: A Systematic Review and Meta-analysis. Environ Health Perspect. 2017 Aug 3;125(8):086001. doi: 10.1289/EHP1632. PMID: 28799918; PMCID: PMC5783655.

Lee YJ. Potential health effects of emerging environmental contaminants perfluoroalkyl compounds. Yeungnam Univ J Med. 2018 Dec;35(2):156-164. doi: 10.12701/yujm.2018.35.2.156. Epub 2018 Dec 31. PMID: 31620588; PMCID: PMC6784697.

Nilsen FM, Tulve NS. A systematic review and meta-analysis examining the interrelationships between chemical and non-chemical stressors and inherent characteristics in children with ADHD. Environ Res. 2020 Jan;180:108884. doi: 10.1016/j.envres.2019.108884. Epub 2019 Nov 1. PMID: 31706600; PMCID: PMC6937727.

Praveena SM, Munisvaradass R, Masiran R, Rajendran RK, Lin CC, Kumar S. Phthalates exposure and attention-deficit/hyperactivity disorder in children: a systematic review of epidemiological literature. Environ Sci Pollut Res Int. 2020 Dec;27(36):44757-44770. doi: 10.1007/s11356-020-10652-z. Epub 2020 Sep 7. PMID: 32895790.

Rezaei Kalantary R, Jaffarzadeh N, Rezapour M, Hesami Arani M. Association between exposure to polycyclic aromatic hydrocarbons and attention deficit hyperactivity disorder in children: a systematic review and meta-analysis. Environ Sci Pollut Res Int. 2020 Apr;27(11):11531-11540. doi: 10.1007/s11356-020-08134-3. Epub 2020 Mar 2. PMID: 32124297.

Roberts JR, Dawley EH, Reigart JR. Children's low-level pesticide exposure and associations with autism and ADHD: a review. Pediatr Res. 2019 Jan;85(2):234-241. doi: 10.1038/s41390-018-0200-z. Epub 2018 Oct 8. PMID: 30337670.

Rochester JR, Bolden AL, Kwiatkowski CF. Prenatal exposure to bisphenol A and hyperactivity in children: a systematic review and meta-analysis. Environ Int. 2018 May;114:343-356. doi: 10.1016/j.envint.2017.12.028. Epub 2018 Mar 7. PMID: 29525285.

Sanders AP, Claus Henn B, Wright RO. Perinatal and Childhood Exposure to Cadmium, Manganese, and Metal Mixtures and Effects on Cognition and Behavior: A Review of Recent Literature. Curr Environ Health Rep. 2015 Sep;2(3):284-94. doi: 10.1007/s40572-015-0058-8. PMID: 26231505; PMCID: PMC4531257.

Tessari L, Angriman M, Díaz-Román A, Zhang J, Conca A, Cortese S. Association Between Exposure to Pesticides and ADHD or Autism Spectrum Disorder: A Systematic Review of the Literature. J Atten Disord. 2022 Jan;26(1):48-71. doi: 10.1177/1087054720940402. Epub 2020 Jul 22. PMID: 32697136.

Yoshimasu K, Kiyohara C, Takemura S, Nakai K. A meta-analysis of the evidence on the impact of prenatal and early infancy exposures to mercury on autism and attention deficit/hyperactivity disorder in the childhood. Neurotoxicology. 2014 Sep;44:121-31. doi: 10.1016/j.neuro.2014.06.007. Epub 2014 Jun 19. PMID: 24952233.

Individual papers listed by substance

Lead (Pb)

Boucher O, Jacobson SW, Plusquellec P, Dewailly E, Ayotte P, Forget-Dubois N, Jacobson JL, Muckle G. Prenatal methylmercury, postnatal lead exposure, and evidence of attention deficit/hyperactivity disorder among Inuit children in Arctic Québec. Environ Health Perspect. 2012 Oct;120(10):1456-61. doi: 10.1289/ehp.1204976. Epub 2012 Sep 21. PMID: 23008274; PMCID: PMC3491943.

Chan TJ, Gutierrez C, Ogunseitan OA. Metallic Burden of Deciduous Teeth and Childhood Behavioral Deficits. Int J Environ Res Public Health. 2015 Jun 15;12(6):6771-87. doi: 10.3390/ijerph120606771. PMID: 26084001; PMCID: PMC4483729.

Choi WJ, Kwon HJ, Lim MH, Lim JA, Ha M. Blood lead, parental marital status and the risk of attention-deficit/hyperactivity disorder in elementary school children: A longitudinal study. Psychiatry Res. 2016 Feb 28;236:42-46. doi: 10.1016/j.psychres.2016.01.002. Epub 2016 Jan 6. PMID: 26774190.

Forns J, Fort M, Casas M, Cáceres A, Guxens M, Gascon M, Garcia-Esteban R, Julvez J, Grimalt JO, Sunyer J. Exposure to metals during pregnancy and neuropsychological development at the age of 4 years. Neurotoxicology. 2014 Jan;40:16-22. doi: 10.1016/j.neuro.2013.10.006. Epub 2013 Nov 6. PMID: 24211492.

Ha M, Kwon HJ, Lim MH, Jee YK, Hong YC, Leem JH, Sakong J, Bae JM, Hong SJ, Roh YM, Jo SJ. Low blood levels of lead and mercury and symptoms of attention deficit hyperactivity in children: a report of the children's health and environment research (CHEER). Neurotoxicology. 2009 Jan;30(1):31-6. doi: 10.1016/j.neuro.2008.11.011. Epub 2008 Nov 30. PMID: 19100765.

Hong SB, Im MH, Kim JW, Park EJ, Shin MS, Kim BN, Yoo HJ, Cho IH, Bhang SY, Hong YC, Cho SC. Environmental lead exposure and attention deficit/hyperactivity disorder symptom domains in a community sample of South Korean school-age children. Environ Health Perspect. 2015 Mar;123(3):271-6. doi: 10.1289/ehp.1307420. Epub 2014 Oct 3. PMID: 25280233; PMCID: PMC4348739.

Huang S, Hu H, Sánchez BN, Peterson KE, Ettinger AS, Lamadrid-Figueroa H, Schnaas L, Mercado-García A, Wright RO, Basu N, Cantonwine DE, Hernández-Avila M, Téllez-Rojo MM. Childhood Blood Lead Levels and Symptoms of Attention Deficit Hyperactivity Disorder (ADHD): A Cross-Sectional Study of Mexican Children. Environ Health Perspect. 2016 Jun;124(6):868-74. doi: 10.1289/ehp.1510067. Epub 2015 Dec 8. PMID: 26645203; PMCID: PMC4892926.

Ji Y, Hong X, Wang G, Chatterjee N, Riley AW, Lee LC, Surkan PJ, Bartell TR, Zuckerman B, Wang X. A Prospective Birth Cohort Study on Early Childhood Lead Levels and Attention Deficit Hyperactivity Disorder: New Insight on Sex Differences. J Pediatr. 2018 Aug;199:124-131.e8. doi: 10.1016/j.jpeds.2018.03.076. Epub 2018 May 8. PMID: 29752174; PMCID: PMC6063774.

Joo H, Lim MH, Ha M, Kwon HJ, Yoo SJ, Choi KH, Paik KC. Secondhand Smoke Exposure and Low Blood Lead Levels in Association With Attention-Deficit Hyperactivity Disorder and Its Symptom Domain in Children: A Community-Based Case-Control Study. Nicotine Tob Res. 2017 Jan;19(1):94-101. doi: 10.1093/ntr/ntw152. Epub 2016 Jul 7. PMID: 27613950; PMCID: PMC5157713.

Kim S, Arora M, Fernandez C, Landero J, Caruso J, Chen A. Lead, mercury, and cadmium exposure and attention deficit hyperactivity disorder in children. Environ Res. 2013 Oct;126:105-10. doi: 10.1016/j.envres.2013.08.008. Epub 2013 Sep 10. PMID: 24034783; PMCID: PMC3847899.

Dikme G, Arvas A, Gür E. The relation between blood lead and mercury levels and chronic neurological diseases in children. Turk Arch Pediatr. 2013; 48: 221-225 DOI: 10.4274/tpa.296

Kim Y, Cho SC, Kim BN, Hong YC, Shin MS, Yoo HJ, Kim JW, Bhang SY. Association between blood lead levels (<5 μg/dL) and inattention-hyperactivity and neurocognitive profiles in school-aged Korean children. Sci Total Environ. 2010 Nov 1;408(23):5737-43. doi: 10.1016/j.scitotenv.2010.07.070. Epub 2010 Sep 9. PMID: 20825975.

Lee MJ, Chou MC, Chou WJ, Huang CW, Kuo HC, Lee SY, Wang LJ. Heavy Metals' Effect on Susceptibility to Attention-Deficit/Hyperactivity Disorder: Implication of Lead, Cadmium, and Antimony. Int J Environ Res Public Health. 2018 Jun 10;15(6):1221. doi: 10.3390/ijerph15061221. PMID: 29890770; PMCID: PMC6025252.

Liu J, Liu X, Wang W, McCauley L, Pinto-Martin J, Wang Y, Li L, Yan C, Rogan WJ. Blood lead concentrations and children's behavioral and emotional problems: a cohort study. JAMA Pediatr. 2014 Aug;168(8):737-45. doi: 10.1001/jamapediatrics.2014.332. PMID: 25090293; PMCID: PMC4152857.

Neugebauer J, Wittsiepe J, Kasper-Sonnenberg M, Schöneck N, Schölmerich A, Wilhelm M. The influence of low level pre- and perinatal exposure to PCDD/Fs, PCBs, and lead on attention performance and attention-related behavior among German school-aged children: results from the Duisburg Birth Cohort Study. Int J Hyg Environ Health. 2015 Jan;218(1):153-62. doi: 10.1016/j.ijheh.2014.09.005. Epub 2014 Oct 5. PMID: 25456149.

Nicolescu R, Petcu C, Cordeanu A, Fabritius K, Schlumpf M, Krebs R, Krämer U, Winneke G. Environmental exposure to lead, but not other neurotoxic metals, relates to core elements of ADHD in Romanian children: performance and questionnaire data. Environ Res. 2010 Jul;110(5):476-83. doi: 10.1016/j.envres.2010.04.002. PMID: 20434143.

Nigg JT, Nikolas M, Mark Knottnerus G, Cavanagh K, Friderici K. Confirmation and extension of association of blood lead with attention-deficit/hyperactivity disorder (ADHD) and ADHD symptom domains at population-typical exposure levels. J Child Psychol Psychiatry. 2010 Jan;51(1):58-65. doi: 10.1111/j.1469-7610.2009.02135.x. Epub 2009 Nov 23. PMID: 19941632; PMCID: PMC2810427.

Park JH, Seo JH, Hong YS, Kim YM, Kang JW, Yoo JH, Chueh HW, Lee JH, Kwak MJ, Kim J, Woo HD, Kim DW, Bang YR, Choe BM. Blood lead concentrations and attention deficit hyperactivity disorder in Korean children: a hospital-based case control study. BMC Pediatr. 2016 Sep 22;16(1):156. doi: 10.1186/s12887-016-0696-5. PMID: 27659349; PMCID: PMC5034496.

Roy A, Bellinger D, Hu H, Schwartz J, Ettinger AS, Wright RO, Bouchard M, Palaniappan K, Balakrishnan K. Lead exposure and behavior among young children in Chennai, India. Environ Health Perspect. 2009 Oct;117(10):1607-11. doi: 10.1289/ehp.0900625. Epub 2009 Jun 26. PMID: 20019913; PMCID: PMC2790517.

Sioen I, Den Hond E, Nelen V, Van de Mieroop E, Croes K, Van Larebeke N, Nawrot TS, Schoeters G. Prenatal exposure to environmental contaminants and behavioural problems at age 7-8years. Environ Int. 2013 Sep;59:225-31. doi: 10.1016/j.envint.2013.06.014. Epub 2013 Jul 9. PMID: 23845936.

Wang HL, Chen XT, Yang B, Ma FL, Wang S, Tang ML, Hao MG, Ruan DY. Case-control study of blood lead levels and attention deficit hyperactivity disorder in Chinese children. Environ Health Perspect. 2008 Oct;116(10):1401-6. doi: 10.1289/ehp.11400. Epub 2008 Jun 5. PMID: 18941585; PMCID: PMC2569102.

Yang R, Zhang Y, Gao W, Lin N, Li R, Zhao Z. Blood Levels of Trace Elements in Children with Attention-Deficit Hyperactivity Disorder: Results from a Case-Control Study. Biol Trace Elem Res. 2019 Feb;187(2):376-382. doi: 10.1007/s12011-018-1408-9. Epub 2018 Jun 16. PMID: 29909491.

Yousef S, Adem A, Zoubeidi T, Kosanovic M, Mabrouk AA, Eapen V. Attention deficit hyperactivity disorder and environmental toxic metal exposure in the United Arab Emirates. J Trop Pediatr. 2011 Dec;57(6):457-60. doi: 10.1093/tropej/fmq121. Epub 2011 Feb 6. PMID: 21300623.

Yu CJ, Du JC, Chiou HC, Feng CC, Chung MY, Yang W, Chen YS, Chien LC, Hwang B, Chen ML. Sugar-Sweetened Beverage Consumption Is Adversely Associated with Childhood Attention Deficit/Hyperactivity Disorder. Int J Environ Res Public Health. 2016 Jul 4;13(7):678. doi: 10.3390/ijerph13070678. PMID: 27384573; PMCID: PMC4962219.

Yu CJ, Du JC, Chiou HC, Yang SH, Liao KW, Yang W, Chung MY, Chien LC, Hwang B, Chen ML. Attention Deficit/Hyperactivity Disorder and Urinary Nonylphenol Levels: A Case-Control Study in Taiwanese Children. PLoS One. 2016 Feb 18;11(2):e0149558. doi: 10.1371/journal.pone.0149558. PMID: 26890918; PMCID: PMC4758720.

Zhang R, Huo X, Ho G, Chen X, Wang H, Wang T, Ma L. Attention-deficit/hyperactivity symptoms in preschool children from an e-waste recycling town: assessment by the parent report derived from DSM-IV. BMC Pediatr. 2015 May 5;15:51. doi: 10.1186/s12887-015-0368-x. PMID: 25939992; PMCID: PMC4429982.

Phthalates

Arbuckle TE, Davis K, Boylan K, Fisher M, Fu J. Bisphenol A, phthalates and lead and learning and behavioral problems in Canadian children 6-11 years of age: CHMS 2007-2009. Neurotoxicology. 2016 May;54:89-98. doi: 10.1016/j.neuro.2016.03.014. Epub 2016 Mar 25. PMID: 27021348.

Balalian AA, Whyatt RM, Liu X, Insel BJ, Rauh VA, Herbstman J, Factor-Litvak P. Prenatal and childhood exposure to phthalates and motor skills at age 11 years. Environ Res. 2019 Apr;171:416-427. doi: 10.1016/j.envres.2019.01.046. Epub 2019 Jan 29. PMID: 30731329; PMCID: PMC6814270.

Chopra V, Harley K, Lahiff M, Eskenazi B. Association between phthalates and attention deficit disorder and learning disability in U.S. children, 6-15 years. Environ Res. 2014 Jan;128:64-9. doi: 10.1016/j.envres.2013.10.004. Epub 2013 Nov 19. PMID: 24267794; PMCID: PMC3889659.

Engel SM, Miodovnik A, Canfield RL, Zhu C, Silva MJ, Calafat AM, Wolff MS. Prenatal phthalate exposure is associated with childhood behavior and executive functioning. Environ Health Perspect. 2010 Apr;118(4):565-71. doi: 10.1289/ehp.0901470. Epub 2010 Jan 8. PMID: 20106747; PMCID: PMC2854736.

Engel SM, Villanger GD, Nethery RC, Thomsen C, Sakhi AK, Drover SSM, Hoppin JA, Zeiner P, Knudsen GP, Reichborn-Kjennerud T, Herring AH, Aase H. Prenatal Phthalates, Maternal Thyroid Function, and Risk of Attention-Deficit Hyperactivity Disorder in the Norwegian Mother and Child Cohort. Environ Health Perspect. 2018 May 10;126(5):057004. doi: 10.1289/EHP2358. PMID: 29790729; PMCID: PMC6071976.

England-Mason G, Martin JW, MacDonald A, Kinniburgh D, Giesbrecht GF, Letourneau N, Dewey D. Similar names, different results: Consistency of the associations between prenatal exposure to phthalates and parent-ratings of behavior problems in preschool children. Environ Int. 2020 Sep;142:105892. doi: 10.1016/j.envint.2020.105892. Epub 2020 Jun 25. PMID: 32593833; PMCID: PMC7493743.

Hu D, Wang YX, Chen WJ, Zhang Y, Li HH, Xiong L, Zhu HP, Chen HY, Peng SX, Wan ZH, Zhang Y, Du YK. Associations of phthalates exposure with attention deficits hyperactivity disorder: A case-control study among Chinese children. Environ Pollut. 2017 Oct;229:375-385. doi: 10.1016/j.envpol.2017.05.089. Epub 2017 Jun 12. PMID: 28614761.

Kim BN, Cho SC, Kim Y, Shin MS, Yoo HJ, Kim JW, Yang YH, Kim HW, Bhang SY, Hong YC. Phthalates exposure and attention-deficit/hyperactivity disorder in school-age children. Biol Psychiatry. 2009 Nov 15;66(10):958-63. doi: 10.1016/j.biopsych.2009.07.034. Epub 2009 Sep 12. PMID: 19748073.

Kobrosly RW, Evans S, Miodovnik A, Barrett ES, Thurston SW, Calafat AM, Swan SH. Prenatal phthalate exposures and neurobehavioral development scores in boys and girls at 6-10 years of age. Environ Health Perspect. 2014 May;122(5):521-8. doi: 10.1289/ehp.1307063. Epub 2014 Feb 21. PMID: 24577876; PMCID: PMC4014764.

Ku HY, Tsai TL, Wang PL, Su PH, Sun CW, Wang CJ, Wang SL. Prenatal and childhood phthalate exposure and attention deficit hyperactivity disorder traits in child temperament: A 12-year follow-up birth cohort study. Sci Total Environ. 2020 Jan 10;699:134053. doi: 10.1016/j.scitotenv.2019.134053. Epub 2019 Aug 29. PMID: 31678884.

Lien YJ, Ku HY, Su PH, Chen SJ, Chen HY, Liao PC, Chen WJ, Wang SL. Prenatal exposure to phthalate esters and behavioral syndromes in children at 8 years of age: Taiwan Maternal and Infant Cohort Study. Environ Health Perspect. 2015 Jan;123(1):95-100. doi: 10.1289/ehp.1307154. Epub 2014 Oct 3. PMID: 25280125; PMCID: PMC4286269.

Messerlian C, Bellinger D, Mínguez-Alarcón L, Romano ME, Ford JB, Williams PL, Calafat AM, Hauser R, Braun JM. Paternal and maternal preconception urinary phthalate metabolite concentrations and child behavior. Environ Res. 2017 Oct;158:720-728. doi: 10.1016/j.envres.2017.07.032. Epub 2017 Jul 21. PMID: 28738300; PMCID: PMC5599166.

Minatoya M, Itoh S, Yamazaki K, Araki A, Miyashita C, Tamura N, Yamamoto J, Onoda Y, Ogasawara K, Matsumura T, Kishi R. Prenatal exposure to bisphenol A and phthalates and behavioral problems in children at preschool age: the Hokkaido Study on Environment and Children's Health. Environ Health Prev Med. 2018 Sep 7;23(1):43. doi: 10.1186/s12199-018-0732-1. PMID: 30193567; PMCID: PMC6129008.

Park S, Lee JM, Kim JW, Cheong JH, Yun HJ, Hong YC, Kim Y, Han DH, Yoo HJ, Shin MS, Cho SC, Kim BN. Association between phthalates and externalizing behaviors and cortical thickness in children with attention deficit hyperactivity disorder. Psychol Med. 2015 Jun;45(8):1601-12. doi: 10.1017/S0033291714002694. Epub 2014 Nov 12. PMID: 25388623.

Verstraete S, Vanhorebeek I, Covaci A, Güiza F, Malarvannan G, Jorens PG, Van den Berghe G. Circulating phthalates during critical illness in children are associated with long-term attention deficit: a study of a development and a validation cohort. Intensive Care Med. 2016 Mar;42(3):379-392. doi: 10.1007/s00134-015-4159-5. Epub 2015 Dec 14. PMID: 26667027.

Won EK, Kim Y, Ha M, Burm E, Kim YS, Lim H, Jung DE, Lim S, Kim SY, Kim YM, Kim HC, Lee KJ, Cheong HK, Kang HT, Son M, Sakong J, Oh GJ, Lee CG, Kim SY, Ryu JM, Kim SJ. Association of current phthalate exposure with neurobehavioral development in a national sample. Int J Hyg Environ Health. 2016 Jul;219(4-5):364-71. doi: 10.1016/j.ijheh.2016.03.001. Epub 2016 Mar 3. PMID: 26987946.

Bisphenol A (BPA)

Braun JM, Kalkbrenner AE, Calafat AM, Yolton K, Ye X, Dietrich KN, Lanphear BP. Impact of early-life bisphenol A exposure on behavior and executive function in children. Pediatrics. 2011 Nov;128(5):873-82. doi: 10.1542/peds.2011-1335. Epub 2011 Oct 24. PMID: 22025598; PMCID: PMC3208956.

Casas M, Forns J, Martínez D, Avella-García C, Valvi D, Ballesteros-Gómez A, Luque N, Rubio S, Julvez J, Sunyer J, Vrijheid M. Exposure to bisphenol A during pregnancy and child neuropsychological development in the INMA-Sabadell cohort. Environ Res. 2015 Oct;142:671-9. doi: 10.1016/j.envres.2015.07.024. PMID: 26343751.

Harley KG, Gunier RB, Kogut K, Johnson C, Bradman A, Calafat AM, Eskenazi B. Prenatal and early childhood bisphenol A concentrations and behavior in school-aged children. Environ Res. 2013 Oct;126:43-50. doi: 10.1016/j.envres.2013.06.004. Epub 2013 Jul 17. PMID: 23870093; PMCID: PMC3805756.

Li Y, Zhang H, Kuang H, Fan R, Cha C, Li G, Luo Z, Pang Q. Relationship between bisphenol A exposure and attention-deficit/ hyperactivity disorder: A case-control study for primary school children in Guangzhou, China. Environ Pollut. 2018 Apr;235:141-149. doi: 10.1016/ j.envpol.2017.12.056. Epub 2017 Dec 23. PMID: 29276960.

Tewar S, Auinger P, Braun JM, Lanphear B, Yolton K, Epstein JN, Ehrlich S, Froehlich TE. Association of Bisphenol A exposure and Attention-Deficit/ Hyperactivity Disorder in a national sample of U.S. children. Environ Res. 2016 Oct;150:112-118. doi: 10.1016/j.envres.2016.05.040. Epub 2016 Jun 6. PMID: 27281688.

Polycyclic aromatic hydrocarbons (PAHs)

Abid Z, Roy A, Herbstman JB, Ettinger AS. Urinary polycyclic aromatic hydrocarbon metabolites and attention/deficit hyperactivity disorder, learning disability, and special education in U.S. children aged 6 to 15. J Environ Public Health. 2014;2014:628508. doi: 10.1155/2014/628508. Epub 2014 Jan 30. PMID: 24624143; PMCID: PMC3929190.

Margolis AE, Herbstman JB, Davis KS, Thomas VK, Tang D, Wang Y, Wang S, Perera FP, Peterson BS, Rauh VA. Longitudinal effects of prenatal exposure to air pollutants on self-regulatory capacities and social competence. J Child Psychol Psychiatry. 2016 Jul;57(7):851-60. doi: 10.1111/jcpp.12548. Epub 2016 Mar 17. PMID: 26989990; PMCID: PMC5333974.

Mortamais M, Pujol J, van Drooge BL, Macià D, Martínez-Vilavella G, Reynes C, Sabatier R, Rivas I, Grimalt J, Forns J, Alvarez-Pedrerol M, Querol X, Sunyer J. Effect of exposure to polycyclic aromatic hydrocarbons on basal ganglia and attention-deficit hyperactivity disorder symptoms in primary school children. Environ Int. 2017 Aug;105:12-19. doi: 10.1016/j.envint.2017.04.011. Epub 2017 May 5. PMID: 28482185.

Perera FP, Wang S, Vishnevetsky J, Zhang B, Cole KJ, Tang D, Rauh V, Phillips DH. Polycyclic aromatic hydrocarbons-aromatic DNA adducts in cord blood and behavior scores in New York city children. Environ Health Perspect. 2011 Aug;119(8):1176-81. doi: 10.1289/ehp.1002705. Epub 2011 Apr 12. PMID: 21486719; PMCID: PMC3237340.

Perera FP, Tang D, Wang S, Vishnevetsky J, Zhang B, Diaz D, Camann D, Rauh V. Prenatal polycyclic aromatic hydrocarbon (PAH) exposure and child behavior at age 6-7 years. Environ Health Perspect. 2012 Jun;120(6):921-6. doi: 10.1289/ehp.1104315. Epub 2012 Mar 14. PMID: 22440811; PMCID: PMC3385432.

Perera FP, Chang HW, Tang D, Roen EL, Herbstman J, Margolis A, Huang TJ, Miller RL, Wang S, Rauh V. Early-life exposure to polycyclic aromatic hydrocarbons and ADHD behavior problems. PLoS One. 2014 Nov 5;9(11):e111670. doi: 10.1371/journal.pone.0111670. PMID: 25372862; PMCID: PMC4221082.

Perera FP, Wheelock K, Wang Y, Tang D, Margolis AE, Badia G, Cowell W, Miller RL, Rauh V, Wang S, Herbstman JB. Combined effects of prenatal exposure to polycyclic aromatic hydrocarbons and material hardship on child ADHD behavior problems. Environ Res. 2018 Jan;160:506-513. doi: 10.1016/j.envres.2017.09.002. Epub 2017 Oct 4. PMID: 28987706; PMCID: PMC5724364.

Flame retardants

Adgent MA, Hoffman K, Goldman BD, Sjödin A, Daniels JL. Brominated flame retardants in breast milk and behavioural and cognitive development at 36 months. Paediatr Perinat Epidemiol. 2014 Jan;28(1):48-57. doi: 10.1111/ppe.12078. Epub 2013 Aug 19. PMID: 24313667; PMCID: PMC3997742.

Castorina R, Butt C, Stapleton HM, Avery D, Harley KG, Holland N, Eskenazi B, Bradman A. Flame retardants and their metabolites in the homes and urine of pregnant women residing in California (the CHAMACOS cohort). Chemosphere. 2017 Jul;179:159-166. doi: 10.1016/j.chemosphere.2017.03.076. Epub 2017 Mar 22. PMID: 28365501; PMCID: PMC5491392.

Chen A, Yolton K, Rauch SA, Webster GM, Hornung R, Sjödin A, Dietrich KN, Lanphear BP. Prenatal polybrominated diphenyl ether exposures and neurodevelopment in U.S. children through 5 years of age: the HOME study. Environ Health Perspect. 2014 Aug;122(8):856-62. doi: 10.1289/ehp.1307562. Epub 2014 May 28. PMID: 24870060; PMCID: PMC4123029.

Cowell WJ, Lederman SA, Sjödin A, Jones R, Wang S, Perera FP, Wang R, Rauh VA, Herbstman JB. Prenatal exposure to polybrominated diphenyl ethers and child attention problems at 3-7 years. Neurotoxicol Teratol. 2015 Nov-Dec;52(Pt B):143-50. doi: 10.1016/j.ntt.2015.08.009. Epub 2015 Sep 5. PMID: 26344673; PMCID: PMC4785171.

Doherty BT, Hoffman K, Keil AP, Engel SM, Stapleton HM, Goldman BD, Olshan AF, Daniels JL. Prenatal exposure to organophosphate esters and behavioral development in young children in the Pregnancy, Infection, and Nutrition Study. Neurotoxicology. 2019 Jul;73:150-160. doi: 10.1016/j.neuro.2019.03.007. Epub 2019 Apr 3. PMID: 30951742; PMCID: PMC6635002.

Eskenazi B, Chevrier J, Rauch SA, Kogut K, Harley KG, Johnson C, Trujillo C, Sjödin A, Bradman A. In utero and childhood polybrominated diphenyl ether (PBDE) exposures and neurodevelopment in the CHAMACOS study. Environ Health Perspect. 2013 Feb;121(2):257-62. doi: 10.1289/ehp.1205597. Epub 2012 Nov 15. PMID: 23154064; PMCID: PMC3569691.

Gascon M, Vrijheid M, Martínez D, Forns J, Grimalt JO, Torrent M, Sunyer J. Effects of pre and postnatal exposure to low levels of polybromodiphenyl ethers on neurodevelopment and thyroid hormone levels at 4 years of age. Environ Int. 2011 Apr;37(3):605-11. doi: 10.1016/j.envint.2010.12.005. Epub 2011 Jan 14. PMID: 21237513.

Gump BB, Yun S, Kannan K. Polybrominated diphenyl ether (PBDE) exposure in children: possible associations with cardiovascular and psychological functions. Environ Res. 2014 Jul;132:244-50. doi: 10.1016/j.envres.2014.04.009. Epub 2014 May 13. PMID: 24834818; PMCID: PMC4104497.

Hoffman K, Adgent M, Goldman BD, Sjödin A, Daniels JL. Lactational exposure to polybrominated diphenyl ethers and its relation to social and emotional development among toddlers. Environ Health Perspect. 2012 Oct;120(10):1438-42. doi: 10.1289/ehp.1205100. Epub 2012 Jul 19. PMID: 22814209; PMCID: PMC3491946.

Roze E, Meijer L, Bakker A, Van Braeckel KN, Sauer PJ, Bos AF. Prenatal exposure to organohalogens, including brominated flame retardants, influences motor, cognitive, and behavioral performance at school age. Environ Health Perspect. 2009 Dec;117(12):1953-8. doi: 10.1289/ehp.0901015. Epub 2009 Aug 31. PMID: 20049217; PMCID: PMC2799472.

Sagiv SK, Kogut K, Gaspar FW, Gunier RB, Harley KG, Parra K, Villaseñor D, Bradman A, Holland N, Eskenazi B. Prenatal and childhood polybrominated diphenyl ether (PBDE) exposure and attention and executive function at 9-12 years of age. Neurotoxicol Teratol. 2015 Nov-Dec;52(Pt B):151-61. doi: 10.1016/j.ntt.2015.08.001. Epub 2015 Aug 10. PMID: 26271888; PMCID: PMC5072748.

Mercury (Hg)

Boucher O, Jacobson SW, Plusquellec P, Dewailly E, Ayotte P, Forget-Dubois N, Jacobson JL, Muckle G. Prenatal methylmercury, postnatal lead exposure, and evidence of attention deficit/hyperactivity disorder among Inuit children in Arctic Québec. Environ Health Perspect. 2012 Oct;120(10):1456-61. doi: 10.1289/ehp.1204976. Epub 2012 Sep 21. PMID: 23008274; PMCID: PMC3491943.

Ha M, Kwon HJ, Lim MH, Jee YK, Hong YC, Leem JH, Sakong J, Bae JM, Hong SJ, Roh YM, Jo SJ. Low blood levels of lead and mercury and symptoms of attention deficit hyperactivity in children: a report of the children's health and environment research (CHEER). Neurotoxicology. 2009 Jan;30(1):31-6. doi: 10.1016/j.neuro.2008.11.011. Epub 2008 Nov 30. PMID: 19100765.

Sagiv SK, Thurston SW, Bellinger DC, Amarasiriwardena C, Korrick SA. Prenatal exposure to mercury and fish consumption during pregnancy and attention-deficit/hyperactivity disorder-related behavior in children. Arch Pediatr Adolesc Med. 2012 Dec;166(12):1123-31. doi: 10.1001/archpediatrics.2012.1286. PMID: 23044994; PMCID: PMC3991460.

Yousef S, Adem A, Zoubeidi T, Kosanovic M, Mabrouk AA, Eapen V. Attention deficit hyperactivity disorder and environmental toxic metal exposure in the United Arab Emirates. J Trop Pediatr. 2011 Dec;57(6):457-60. doi: 10.1093/tropej/fmq121. Epub 2011 Feb 6. PMID: 21300623.

Pesticides

Bouchard MF, Bellinger DC, Wright RO, Weisskopf MG. Attention-deficit/hyperactivity disorder and urinary metabolites of organophosphate pesticides. Pediatrics. 2010 Jun;125(6):e1270-7. doi: 10.1542/peds.2009-3058. Epub 2010 May 17. PMID: 20478945; PMCID: PMC3706632.

Eskenazi B, Marks AR, Bradman A, Harley K, Barr DB, Johnson C, Morga N, Jewell NP. Organophosphate pesticide exposure and neurodevelopment in young Mexican-American children. Environ Health Perspect. 2007 May;115(5):792-8. doi: 10.1289/ehp.9828. Epub 2007 Jan 4. PMID: 17520070; PMCID: PMC1867968.

Forns J, Stigum H, Høyer BB, Sioen I, Sovcikova E, Nowack N, Lopez-Espinosa MJ, Guxens M, Ibarluzea J, Torrent M, Wittsiepe J, Govarts E, Trnovec T, Chevrier C, Toft G, Vrijheid M, Iszatt N, Eggesbø M. Prenatal and postnatal exposure to persistent organic pollutants and attention-deficit and hyperactivity disorder: a pooled analysis of seven European birth cohort studies. Int J Epidemiol. 2018 Aug 1;47(4):1082-1097. doi: 10.1093/ije/dyy052. PMID: 29912347; PMCID: PMC6124627.

Fortenberry GZ, Meeker JD, Sánchez BN, Barr DB, Panuwet P, Bellinger D, Schnaas L, Solano-González M, Ettinger AS, Hernandez-Avila M, Hu H, Tellez-Rojo MM. Urinary 3,5,6-trichloro-2-pyridinol (TCPY) in pregnant women from Mexico City: distribution, temporal variability, and relationship with child attention and hyperactivity. Int J Hyg Environ Health. 2014 Mar;217(2-3):405-12. doi: 10.1016/j.ijheh.2013.07.018. Epub 2013 Aug 13. PMID: 24001412; PMCID: PMC3946926.

Lenters V, Iszatt N, Forns J, Čechová E, Kočan A, Legler J, Leonards P, Stigum H, Eggesbø M. Early-life exposure to persistent organic pollutants (OCPs, PBDEs, PCBs, PFASs) and attention-deficit/hyperactivity disorder: A multi-pollutant analysis of a Norwegian birth cohort. Environ Int. 2019 Apr;125:33-42. doi: 10.1016/j.envint.2019.01.020. Epub 2019 Jan 28. PMID: 30703609.

Marks AR, Harley K, Bradman A, Kogut K, Barr DB, Johnson C, Calderon N, Eskenazi B. Organophosphate pesticide exposure and attention in young Mexican-American children: the CHAMACOS study. Environ Health Perspect. 2010 Dec;118(12):1768-74. doi: 10.1289/ehp.1002056. PMID: 21126939; PMCID: PMC3002198.

Newman J, Behforooz B, Khuzwayo AG, Gallo MV, Schell LM; Akwesasne Task Force on the Environment. PCBs and ADHD in Mohawk adolescents. Neurotoxicol Teratol. 2014 Mar-Apr;42:25-34. doi: 10.1016/j.ntt.2014.01.005. Epub 2014 Jan 21. PMID: 24462617; PMCID: PMC4907323.

Oulhote Y, Bouchard MF. Urinary metabolites of organophosphate and pyrethroid pesticides and behavioral problems in Canadian children. Environ Health Perspect. 2013 Nov-Dec;121(11-12):1378-84. doi: 10.1289/ehp.1306667. Epub 2013 Oct 22. PMID: 24149046; PMCID: PMC3855516.

Quirós-Alcalá L, Mehta S, Eskenazi B. Pyrethroid pesticide exposure and parental report of learning disability and attention deficit/hyperactivity disorder in U.S. children: NHANES 1999-2002. Environ Health Perspect. 2014 Dec;122(12):1336-42. doi: 10.1289/ehp.1308031. Epub 2014 Sep 5. PMID: 25192380; PMCID: PMC4256700.

Saez M, Barceló MA, Farrerons M, López-Casasnovas G. The association between exposure to environmental factors and the occurrence of attention-deficit/hyperactivity disorder (ADHD). A population-based retrospective cohort study. Environ Res. 2018 Oct;166:205-214. doi: 10.1016/j.envres.2018.05.009. Epub 2018 Jun 15. PMID: 29890425.

Sagiv SK, Thurston SW, Bellinger DC, Tolbert PE, Altshul LM, Korrick SA. Prenatal organochlorine exposure and behaviors associated with attention deficit hyperactivity disorder in school-aged children. Am J Epidemiol. 2010 Mar 1;171(5):593-601. doi: 10.1093/aje/kwp427. Epub 2010 Jan 27. PMID: 20106937; PMCID: PMC2842227.

Sioen I, Den Hond E, Nelen V, Van de Mieroop E, Croes K, Van Larebeke N, Nawrot TS, Schoeters G. Prenatal exposure to environmental contaminants and behavioural problems at age 7-8years. Environ Int. 2013 Sep;59:225-31. doi: 10.1016/j.envint.2013.06.014. Epub 2013 Jul 9. PMID: 23845936.

Rauh VA, Garfinkel R, Perera FP, Andrews HF, Hoepner L, Barr DB, Whitehead R, Tang D, Whyatt RW. Impact of prenatal chlorpyrifos exposure on neurodevelopment in the first 3 years of life among inner-city children. Pediatrics. 2006 Dec;118(6):e1845-59. doi: 10.1542/peds.2006-0338. Epub 2006 Nov 20. PMID: 17116700; PMCID: PMC3390915.

Rauh VA, Garcia WE, Whyatt RM, Horton MK, Barr DB, Louis ED. Prenatal exposure to the organophosphate pesticide chlorpyrifos and childhood tremor. Neurotoxicology. 2015 Dec;51:80-6. doi: 10.1016/j.neuro.2015.09.004. Epub 2015 Sep 15. PMID: 26385760; PMCID: PMC4809635.

Ribas-Fitó N, Torrent M, Carrizo D, Júlvez J, Grimalt JO, Sunyer J. Exposure to hexachlorobenzene during pregnancy and children's social behavior at 4 years of age. Environ Health Perspect. 2007 Mar;115(3):447-50. doi: 10.1289/ehp.9314. Epub 2006 Nov 6. PMID: 17431497; PMCID: PMC1849941.

Richardson JR, Taylor MM, Shalat SL, Guillot TS 3rd, Caudle WM, Hossain MM, Mathews TA, Jones SR, Cory-Slechta DA, Miller GW. Developmental pesticide exposure reproduces features of attention deficit hyperactivity disorder. FASEB J. 2015 May;29(5):1960-72. doi: 10.1096/fj.14-260901. Epub 2015 Jan 28. PMID: 25630971; PMCID: PMC4415012.

van Wendel de Joode B, Mora AM, Lindh CH, Hernández-Bonilla D, Córdoba L, Wesseling C, Hoppin JA, Mergler D. Pesticide exposure and neurodevelopment in children aged 6-9 years from Talamanca, Costa Rica. Cortex. 2016 Dec;85:137-150. doi: 10.1016/j.cortex.2016.09.003. Epub 2016 Sep 15. PMID: 27773359.

Wagner-Schuman M, Richardson JR, Auinger P, Braun JM, Lanphear BP, Epstein JN, Yolton K, Froehlich TE. Association of pyrethroid pesticide exposure with attention-deficit/hyperactivity disorder in a nationally representative sample of U.S. children. Environ Health. 2015 May 28;14:44. doi: 10.1186/s12940-015-0030-y. PMID: 26017680; PMCID: PMC4458051.

Xu X, Nembhard WN, Kan H, Kearney G, Zhang ZJ, Talbott EO. Urinary trichlorophenol levels and increased risk of attention deficit hyperactivity disorder among US school-aged children. Occup Environ Med. 2011 Aug;68(8):557-61. doi: 10.1136/oem.2010.063859. Epub 2011 May 3. PMID: 21540483; PMCID: PMC3131187.

Yu CJ, Du JC, Chiou HC, Chung MY, Yang W, Chen YS, Fuh MR, Chien LC, Hwang B, Chen ML. Increased risk of attention-deficit/hyperactivity disorder associated with exposure to organophosphate pesticide in Taiwanese children. Andrology. 2016 Jul;4(4):695-705. doi: 10.1111/andr.12183. Epub 2016 Apr 12. PMID: 27070915.

Cadmium (Cd)

Ciesielski T, Weuve J, Bellinger DC, Schwartz J, Lanphear B, Wright RO. Cadmium exposure and neurodevelopmental outcomes in U.S. children. Environ Health Perspect. 2012 May;120(5):758-63. doi: 10.1289/ehp.1104152. Epub 2012 Jan 27. PMID: 22289429; PMCID: PMC3346779.

Forns J, Fort M, Casas M, Cáceres A, Guxens M, Gascon M, Garcia-Esteban R, Julvez J, Grimalt JO, Sunyer J. Exposure to metals during pregnancy and neuropsychological development at the age of 4 years. Neurotoxicology. 2014 Jan;40:16-22. doi: 10.1016/j.neuro.2013.10.006. Epub 2013 Nov 6. PMID: 24211492.

Kim S, Arora M, Fernandez C, Landero J, Caruso J, Chen A. Lead, mercury, and cadmium exposure and attention deficit hyperactivity disorder in children. Environ Res. 2013 Oct;126:105-10. doi: 10.1016/j.envres.2013.08.008. Epub 2013 Sep 10. PMID: 24034783; PMCID: PMC3847899.

Szkup-Jabłońska M, Karakiewicz B, Grochans E, Jurczak A, Nowak-Starz G, Rotter I, Prokopowicz A. Effects of blood lead and cadmium levels on the functioning of children with behaviour disorders in the family environment. Ann Agric Environ Med. 2012;19(2):241-6. PMID: 22742795.

Yousef S, Adem A, Zoubeidi T, Kosanovic M, Mabrouk AA, Eapen V. Attention deficit hyperactivity disorder and environmental toxic metal exposure in the United Arab Emirates. J Trop Pediatr. 2011 Dec;57(6):457-60. doi: 10.1093/tropej/fmq121. Epub 2011 Feb 6. PMID: 21300623.

Per- and polyfluoroalkyl substances (PFAS)

Hoffman K, Webster TF, Weisskopf MG, Weinberg J, Vieira VM. Exposure to polyfluoroalkyl chemicals and attention deficit/hyperactivity disorder in U.S. children 12-15 years of age. Environ Health Perspect. 2010 Dec;118(12):1762-7. doi: 10.1289/ehp.1001898. Epub 2010 Jun 15. PMID: 20551004; PMCID: PMC3002197.

Høyer BB, Ramlau-Hansen CH, Obel C, Pedersen HS, Hernik A, Ogniev V, Jönsson BA, Lindh CH, Rylander L, Rignell-Hydbom A, Bonde JP, Toft G. Pregnancy serum concentrations of perfluorinated alkyl substances and offspring behaviour and motor development at age 5-9 years--a prospective study. Environ Health. 2015 Jan 7;14:2. doi: 10.1186/1476-069X-14-2. PMID: 25567242; PMCID: PMC4298045.

Lien GW, Huang CC, Shiu JS, Chen MH, Hsieh WS, Guo YL, Chen PC. Perfluoroalkyl substances in cord blood and attention deficit/hyperactivity disorder symptoms in seven-year-old children. Chemosphere. 2016 Aug;156:118-127. doi: 10.1016/j.chemosphere.2016.04.102. Epub 2016 May 9. PMID: 27174824.

Liew Z, Ritz B, von Ehrenstein OS, Bech BH, Nohr EA, Fei C, Bossi R, Henriksen TB, Bonefeld-Jørgensen EC, Olsen J. Attention deficit/hyperactivity disorder and childhood autism in association with prenatal exposure to perfluoroalkyl substances: a nested case-control study in the Danish National Birth Cohort. Environ Health Perspect. 2015 Apr;123(4):367-73. doi: 10.1289/ehp.1408412. Epub 2014 Dec 19. PMID: 25616253; PMCID: PMC4383573.

Ode A, Källén K, Gustafsson P, Rylander L, Jönsson BA, Olofsson P, Ivarsson SA, Lindh CH, Rignell-Hydbom A. Fetal exposure to perfluorinated compounds and attention deficit hyperactivity disorder in childhood. PLoS One. 2014 Apr 23;9(4):e95891. doi: 10.1371/journal.pone.0095891. PMID: 24760015; PMCID: PMC3997434.

Stein CR, Savitz DA. Serum perfluorinated compound concentration and attention deficit/hyperactivity disorder in children 5-18 years of age. Environ Health Perspect. 2011 Oct;119(10):1466-71. doi: 10.1289/ehp.1003538. Epub 2011 Jun 10. PMID: 21665566; PMCID: PMC3230446.

Stein CR, Savitz DA, Bellinger DC. Perfluorooctanoate exposure in a highly exposed community and parent and teacher reports of behaviour in 6-12-year-old children. Paediatr Perinat Epidemiol. 2014 Mar;28(2):146-56. doi: 10.1111/ppe.12097. Epub 2013 Dec 9. PMID: 24320613; PMCID: PMC4617562.
